# Health literacy and its association with health outcomes among students in upper secondary education: Nina Simonsen

**DOI:** 10.1093/eurpub/ckac131.358

**Published:** 2022-10-25

**Authors:** H Paasio, E Roos, S Kokko, N Simonsen

**Affiliations:** 1 Folkhälsan Research Center, Public Health Research Program, Helsinki, Finland; 2 Uppsala University, Department of Food Studies, Nutrition and Dietetics, Uppsala, Sweden; 3 University of Jyväskylä, Faculty of Sport and Health Sciences, Jyväskylä, Finland; 4 University of Helsinki, Department of Public Health, Helsinki, Finland

## Abstract

**Background:**

Health literacy (HL) - as a broad range of health-related competencies and skills- has been recognized as a determinant of health outcomes, and suggested to be a modifiable health resource and a factor contributing to empowerment and equity. Thus, there is a need to identify the state of HL in various population groups. The aim of this study was to investigate HL levels and associations between HL, physical activity and subjective health among students in general upper secondary and vocational Swedish-language schools in Finland.

**Methods:**

The study used cross-sectional data from the Finnish LIITU-study among students in Swedish-speaking general upper secondary and vocational schools (N = 887; age 16-20 years) conducted in the spring and autumn 2020, during the covid-19-pandemic. Students answered a web-based questionnaire during school hours. HL was measured with the 10-item Health Literacy for School-Aged Children (HLSAC) instrument. Data was analyzed with descriptive statistics and logistic regression analyses.

**Results:**

According to preliminary findings, one third of students had high HL. HL was higher among female than male students, general upper secondary school students than vocational school students, and in spring than in autumn 2020. A higher proportion of students with high HL, as compared to students with low/medium HL, reached the national recommendations for physical activity, perceived their health to be excellent and, moreover, did not have recurrent psychological symptoms.

**Conclusions:**

The findings confirm previous research findings on the importance of HL in promoting health outcomes. The pandemic year 2020 provided a unique perspective to the subject. It would be important to survey and discuss the role of schools in providing equal opportunities for the promotion of HL as this may contribute to decreasing health disparities in the population.

**Key messages:**

• One third of students in upper secondary education (age 16-20 years) had high HL; there were differences between genders, students on different educational paths, and in spring and autumn 2020.

• High HL was associated with better self-rated health, less psychological symptoms and reaching national recommendations for physical activity among students in upper secondary education.

